# Adding a Question About Method Switching to the Method Information Index Is a Better Predictor of Contraceptive Continuation

**DOI:** 10.9745/GHSP-D-19-00028

**Published:** 2019-06-24

**Authors:** Aparna Jain, Kumudha Aruldas, Elizabeth Tobey, Arupendra Mozumdar, Rajib Acharya

**Affiliations:** a Population Council, Washington. DC, USA.; b Population Council, New Delhi, India. Now with Christian Medical College, Vellore, Tamil Nadu, India.; c Population Council, New Delhi, India.

## Abstract

**Introduction:**

The Method Information Index (MII) is 1 of 18 core indicators used to monitor progress toward achieving Family Planning 2020's goal of 120 million more women using a modern method of family planning by 2020. The 3 questions of the MII are intended to measure informed choice at method initiation. Although routinely used in the Demographic and Health Surveys and the Performance Monitoring and Accountability 2020 project in cross-sectional household surveys, the MII may not adequately reflect all key aspects of quality of care or predict contraceptive continuation. In the current study, a question was added to the MII regarding the possibility of switching to a different contraceptive method if the current method is not suitable. The revised MII is referred to as MIIplus.

**Methods:**

A total of 2,699 married women aged 15–49 who started a new episode of use of intrauterine device, injectable, or oral contraceptive pills between December 2016 and October 2017 were followed for 1 year in India and interviewed at method start and 3, 6, and 12 months later. Of these women, 2,267 were interviewed 3 months later and included in the analysis. Using 3 Cox proportional hazard models, we estimated hazard ratios for risk of discontinuation, based on the MII, MIIplus, and a recategorization of MIIplus into a 3-category variable.

**Results:**

The modern method continuation rate 100 days (∼3 months) later was 91% overall. Women who received the information in MIIplus were more likely to continue using a method at 100 days (95%) compared to those who received information covered in the MII (82%) or less than 3 components of the MII (89%) (*P*<.001). Women who received all components in the MIIplus were 69% (adjusted hazard ratio, 0.31; 95% confidence interval: 0.17 to 0.61) less likely than those who received information in the MII to discontinue using a modern method 100 days later. Discontinuation was not significantly different between women who received information on less than the 3 components of the MII compared to the complete MII.

**Conclusion:**

We recommend including the question about the possibility of switching to another family planning method in routine measurement because it better predicts contraceptive continuation than the MII alone and ensures that another domain of quality of care is reflected in the measurement. When programs provide information on the possibility of switching, women are better informed about voluntary family planning choice and their options to continue family planning when a method is not suitable.

## Abstract (precis)

Adding the question “Were you told about the possibility of switching to another method if the method you selected was not suitable?” to the Method Information Index (MII) was associated with better contraceptive continuation. This MIIplus variable includes another domain of quality of care, and thus better reflects voluntary contraceptive use and continuation.

## INTRODUCTION

The Method Information Index (MII) was created as an indicator of informed choice by Family Planning 2020 (FP2020). It is 1 of 18 core indicators used to monitor progress toward achieving the goal of 120 million additional contraceptive users among women with an unmet need for family planning by 2020.^1^ Routinely collected in the Demographic and Health Surveys (DHS) for many years, the MII comprises 3 questions related to women's reports about the information received at the time of method adoption. It is intended to assess the presence of informed choice and includes items from 2 domains of quality of care: (1) information given to clients and solicitation of information from clients for appropriate method selection (information exchange about method selection), and (2) information given to clients by the provider about the method selected (effective use of method selected).^2^

The MII uses the following 3 questions:

1. Were you informed about other methods of family planning?
2. Were you informed about possible side effects or problems you might have with the method?
3. Were you told what to do if you experience any side effects or problems? (asked among those who were told about side effects)

Women who respond yes to all 3 questions are considered to have received full information, based on the MII.

In cross-sectional household surveys, the MII questions are included in the DHS and in the Performance Monitoring and Accountability 2020 (PMA2020) project. In the DHS, the MII questions are asked of current family planning users who adopted the method within 5 years of the survey. In PMA2020 surveys, the MII questions are asked of current and recent users who adopted a method in the past 12 months. MII data have been analyzed to compare differences among regions and countries^1^ and to study variations by female respondents' characteristics and country-level changes over time.^3^

Although these questions have been asked in the DHS for many years, the combination of the 3 questions as the MII is relatively new and thus, much is still unknown about the MII. One question is whether it adequately reflects all 4 domains of quality of care: (1) respectful care; (2) information exchange about method selection; (3) effective use of method chosen; and (4) continuity of contraceptive use and care. Another question is whether receiving all pieces of information covered by the MII relates to contraceptive continuation. Using longitudinal data collected from 2,699 women initiating use of reversible modern contraceptive methods in India, we investigated whether adding the following question to the MII, “Were you told about the possibility of switching to another method if the method you selected was not suitable?”, was associated with a reduced risk of modern method discontinuation 100 days later (∼3 months) compared with receiving information in the MII alone.

We investigated whether adding a question about method switching to the MII reduced the risk of modern method discontinuation.

## METHODS

### Study Areas

The Evidence Project, led by the Population Council, conducted a longitudinal study of married women aged 15–49 in 2 states in India, Haryana and Odisha, who began a new episode of use of a reversible contraceptive method. These 2 states were selected in consultation with the Ministry of Health.

The total fertility rate in India is 2.2 children per woman, and slightly more than 1 in 2 (54%) married women use any type of contraceptive method to space or limit childbearing.^4^ Use of any contraceptive method is greater in Haryana (64%) and Odisha (57%) than in the all-India proportion.^4^ The total fertility rate in Odisha declined from 2.9 children per woman in 1992–1993 to the replacement level of 2.1 children in 2015–2016,^5^ while a more rapid decline was seen in Haryana (4.0 children per women in 1992–1993 to 2.1 in 2015–2016).^6^ In Odisha, 45% of married women between 15 and 49 years old used a modern contraceptive method in 2015–2016,^5^ with the pill being the most popular reversible method (12%). In comparison, Haryana had slightly more users of modern contraceptive (59%), with condoms being the most popular reversible contraceptive method (12%).^6^ Discontinuation of episodes of use within 12 months was 48% for modern reversible methods in Odisha^5^ compared with 41% in Haryana.^6^

This study reflects the current situation related to family planning counseling in the selected areas. No intervention or provider trainings were conducted to improve counseling.

### Data

A new episode of use is defined as a new user of family planning or a past user of family planning who was not using a method right before the method selected at enrollment. These users of the intrauterine device (IUD) (interval and postpartum), injectable, or oral contraceptive pills (OCPs) began a new episode of use between December 2016 and October 2017. They were interviewed within 1 month of the start of this new episode of use (enrollment interview) and at 3, 6, and 12 months following the first interview. The overall purpose of this study was to explore the contraceptive use dynamics of reversible contraceptive users including barriers and facilitators to contraceptive continuation, discontinuation, and method switching. One facilitator/barrier of contraceptive use dynamics explored was quality of care received at the time of method adoption.

Respondents were enrolled from government and private health facilities as well as through accredited social health activists (ASHAs), who are frontline health workers at the community level. In Odisha, at the suggestion of district chief medical officers, postpartum IUD (PPIUD) users were mostly enrolled at selected government health facilities from the labor and delivery wards. In some cases, ASHAs who were identified by the facility managers, enrolled PPIUD users. ASHAs also enrolled interval IUD and OCP users. Injectable users were enrolled at NGO facilities. In Haryana, all respondents were enrolled by ASHAs at the community level.

Face-to-face interviews were conducted at the facility or at the respondent's household, depending on her preference. Study investigators were trained in the study objectives, questionnaires, and informed consent procedures at the beginning of the study and at the start of each follow-up interview. A total of 2,699 women were enrolled in the study and 2,306 were successfully re-interviewed 3 months later, for a loss to follow-up of 14.6%. An additional 23 respondents were excluded from the analysis because they stopped using contraception to become pregnant, and 16 were excluded because of inconsistent dates.

The current study uses data from enrollment and 3-month follow-up interviews for 2 reasons. First, we hypothesized that quality of care received at the time of method adoption would directly affect contraceptive use dynamics in a shorter time frame than a longer one. Second, we hypothesized that women who experience side effects would be more likely to experience them within the first 3 months of use; thus the risk of discontinuation might be greatest during this time period.

The current study uses data from enrollment and 3-month follow-up interviews.

Written consent was obtained from all respondents at the enrollment interview and each follow-up interview. The study received ethical approval from the Population Council Institutional Review Board, the Government of Odisha, and district authorities in selected districts in Haryana.

### Dependent Variable

In this study, the dependent variable is the time in days until the respondent discontinued modern contraceptive use. The observation period is from enrollment until the 3-month follow-up interview or approximately 100 days. All nonusers and traditional method users at the 3-month follow-up interview were considered modern contraceptive discontinuers. Modern contraceptive continuers included users of the method adopted at enrollment as well as method switchers who reported using a modern method at the 3-month follow-up interview that was not the method initiated at enrollment.

### Key Independent Variables

Method information indicators are the key independent variables. They include the MII and method information index plus information received about the possibility of switching methods (MIIplus). In addition, MIIplus was recategorized into a 3-category variable and tested as a separate independent variable.

#### MII

The MII is composed of the 3 questions presented earlier (in the introduction). The MII combines the responses into a dichotomized variable, with women who reported yes to all 3 questions being coded as 1, and women who reported yes to fewer than 3 of these questions being coded as 0.

#### MIIplus

The MIIplus variable adds the question “Were you told about the possibility of switching to another method if the method you selected was not suitable?” to the 3 questions of the MII. This question was asked of all women and not just those who reported receiving information about possible side effects. First, this variable was dichotomized, with women who reported yes to all 4 questions being coded as 1 and women who reported yes on fewer than 4 of these questions being coded as 0.

A 3-category variable disaggregating the 0 category of the MIIplus variable was then constructed to create the MIIplus variation. In this variation, the variable was coded as follows: women who reported yes on fewer than 3 questions of the MII were coded as 0; women who reported yes on all 3 questions of the MII (but did not receive information about the possibility of switching methods) were coded as 1; and women who reported yes on all questions of MIIplus were coded as 2.

### Additional Independent Variables

Several covariates were included in the multivariate models based on theoretical and empirical importance of contraceptive use including age, education, religion, number of living children, method selected at enrollment, previous modern method use, and state.

### Data Analysis

Descriptive statistics were calculated for respondent characteristics and dependent and key independent variables. Survival analysis was applied from the date of enrollment to the date a respondent reported discontinuing a modern contraceptive method. If a respondent was lost to follow-up at the 3-month interview or discontinued using any method because of a desire to become pregnant, the respondent was censored. Kaplan-Meier survival functions were used to estimate continuation rates among the various method information measures. These functions were also used to estimate modern method continuation rates by MIIplus for each method adopted at enrollment. That is, estimates of modern method continuation rates included respondents who were using the same method adopted at enrollment 100 days later and those who switched to another modern method in this period. This shifts the unit of analysis from a specific method to an individual.

Cox proportional hazard models were used to estimate hazard ratios for risk of modern contraceptive discontinuation. Proportional hazard assumptions were checked based on the scaled Schoenfeld residuals. Three hazard models were run, one for each key independent variable. The Akaike information criterion (AIC) was applied to assess model fit between Models I (MII) and II (MIIplus) only because Model III (MIIplus variation) is simply a test of an association, while Models I and II reflect the ways in which the MII and MIIplus are derived. All statistical analyses were conducted in Stata Version 13 (StataCorp, 2013).

## RESULTS

### Respondent Characteristics

Table 1 presents respondent characteristics at enrollment. Most married women enrolled in the study were under the age of 30 (74%), had attended at least primary school (78%), and were Hindu (84%). Nearly all women (99%) had at least 1 child, and a quarter had 3 or more children. Thirty-eight percent had used a modern method previously. In our sample, 40% were OCP users, 39% were IUD users (15% PPIUD and 24% interval IUD), and 21% injectable users.

**TABLE 1.** Respondent Characteristics and Method Information Measures at Enrollment (N=2,699)

Characteristic	No. (%)
**Age, years**	
≤24	1,066 (39.5)
25–29	935 (34.6)
≥30	698 (25.9)
**Education**	
None	605 (22.4)
Primary	335 (12.4)
Middle	386 (14.3)
Secondary	798 (29.6)
Higher secondary	575 (21.3)
**Religion**	
Hindu	2,272 (84.2)
Muslim	418 (15.5)
Other	9 (0.3)
**No. of living children**	
0	23 (0.9)
1	1,118 (41.4)
2	891 (33.0)
3 or more	667 (24.7)
**Previous modern method use**	
Yes	1,037 (38.4)
No	1,662 (61.6)
**Method selected at enrollment**	
OCPs	1,066 (39.5)
PPIUD	412 (15.3)
Interval IUD	640 (23.7)
Injectable	581 (21.5)
**State**	
Haryana	908 (33.6)
Odisha	1,791 (66.4)
**MII**	
<3 questions	1,711 (63.4)
All 3 questions	988 (36.6)
**MIIplus**	
<4 questions	1,779 (65.9)
All 4 questions	920 (34.1)
**MIIplus variation**	
<3 questions of MII	1,711 (63.4)
MII	68 (2.5)
MIIplus	920 (34.1)

Abbreviations: IUD, intrauterine device; MII, Method Information Index; MIIplus, Method Information Index including the question, “Were you told about the possibility of switching to another method if the method you selected was not suitable?”; OCPs, oral contraceptive pills; PPIUD, postpartum intrauterine device.

### Method Information Measures

The distributions of the various method information measures are also presented in Table 1. Thirty-seven percent of respondents received complete information in the MII while 34% received complete information in the MIIplus. When MIIplus was disaggregated into 3 categories (MIIplus variation), only 3% received complete information in the MII.

**FIGURE 1:**
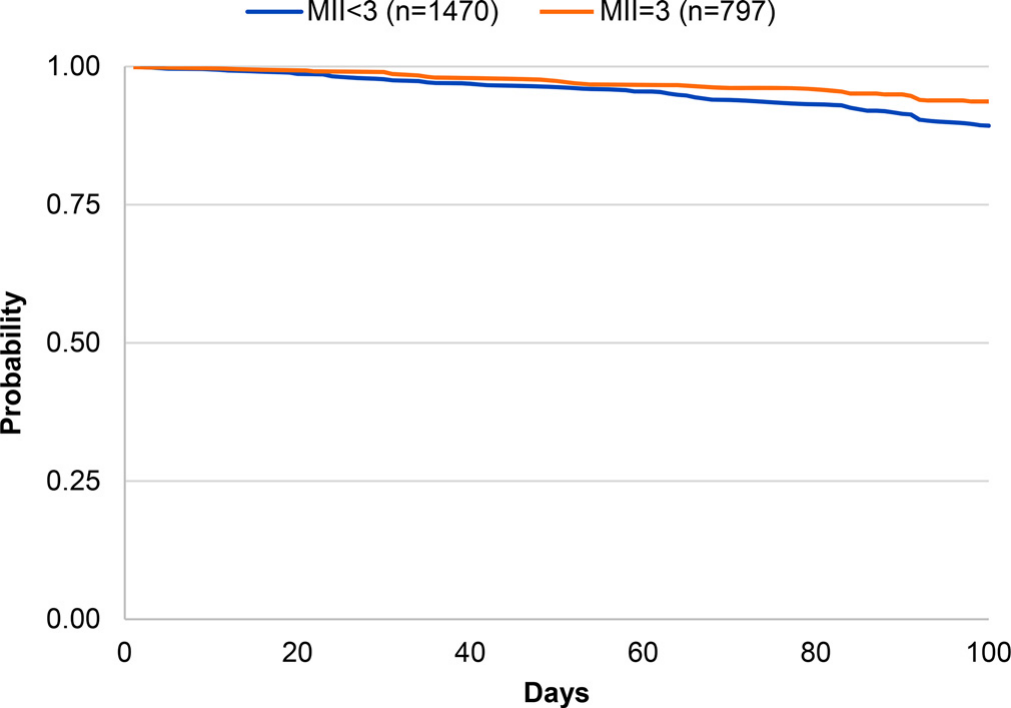
Kaplan-Meier Survival Curves of Contraceptive Continuation by MII Abbreviation: MII, Method Information Index.

**FIGURE 2:**
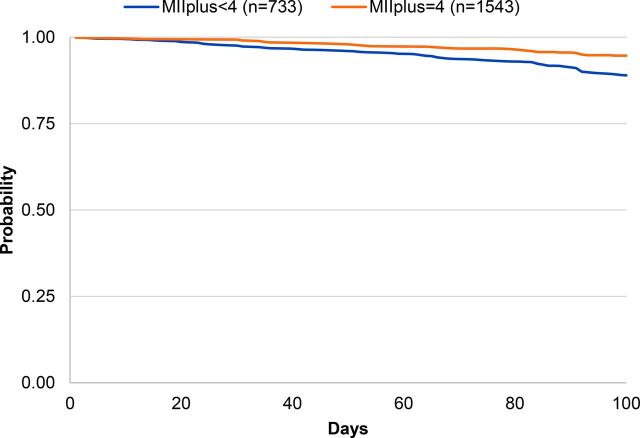
Kaplan-Meier Survival Curves of Contraceptive Continuation by MII With the Added Question About Method Switching (MIIplus) Abbreviation: MII, Method Information Index.

**FIGURE 3:**
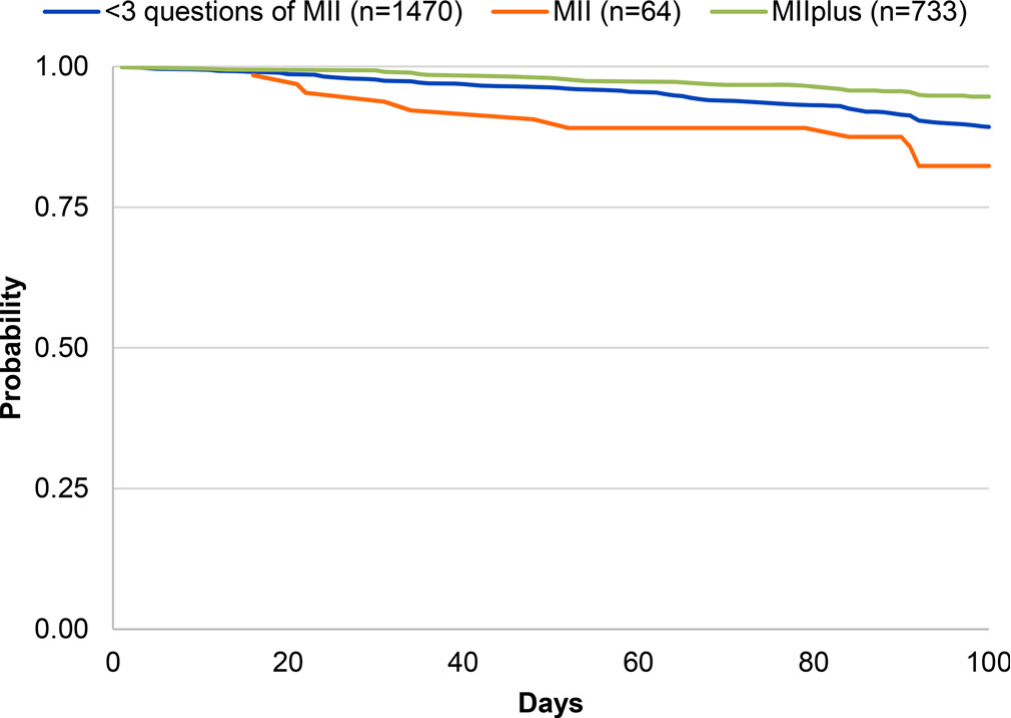
Kaplan-Meier Survival Curves of Contraceptive Continuation by MII With the Added Question About Method Switching (MIIplus) Variation Abbreviation: MII, Method Information Index.

Overall probability of continuing modern method use 100 days later within this sample was 91% (data not shown). Table 2 shows the probability of modern contraceptive continuation at 100 days by the MII, MIIplus, and MIIplus variation. Among respondents who reported receiving complete information in the MII, 94% continued to use a modern contraceptive method 100 days later compared with 89% among those who responded “yes” to fewer than 3 questions of the MII (*P*=.001) (Figure 1). Ninety-five percent of respondents who reported receiving complete information in MIIplus continued to use a modern contraceptive method 100 days later compared with 89% who reported receiving less information (*P*<.001) (Figure 2). Figure 3 shows the Kaplan-Meier survival curves at 100 days for modern contraceptive continuation by the 3 categories of the MIIplus variation. Women who received all information in MIIplus (95%) were more likely to continue using a modern contraceptive at 100 days compared with those who received information covered by the MII (82%) or less than 3 components of the MII (89%) (*P<*.001).

**TABLE 2.** Kaplan-Meier Estimates of 100-Day Continuation of Modern Contraception (n=2,267)

Method Information Indicators	% (95% CI)
**MII**	
<3 questions	89.3 (87.5, 90.8)
All 3 questions	93.7 (91.7, 95.2)
**MIIplus**	
<4 questions	89.0 (87.3, 90.5)
All 4 questions	94.7 (92.7, 96.1)
**MIIplus variation**	
<3 questions of MII	89.3 (87.5, 90.8)
MII	82.3 (70.3, 89.8)
MIIplus	94.7 (92.7, 96.1)

Abbreviations: CI, confidence interval; MII, Method Information Index; MIIplus, Method Information Index including the question, “Were you told about the possibility of switching to another method if the method you selected was not suitable?”

Women who received all information in MIIplus were more likely to still be using a modern contraceptive at 100 days than women who reported receiving less information.

### Method Information Indicators and Risk of Modern Method Discontinuation

Crude and adjusted hazard ratios (HRs) of the risk of contraceptive discontinuation are presented in Table 3. Model I shows that the adjusted HR for discontinuation among women who received complete information based on the MII was 0.65 (95% confidence interval [CI]=0.47 to 0.91) compared with women who received information on less than 3 components, adjusted for age, education, religion, number of living children, previous modern method use, method selected at enrollment, and state. Women who received information on all 4 components of MIIplus were less likely to discontinue 100 days later than those who received information on less than the 4 components (Model II adjusted HR=0.53; 95% CI=0.37 to 0.76). The AIC for MII (Model I) was higher than the AIC for MIIplus (Model II), suggesting a better fit of the data for MIIplus.

**TABLE 3.** Crude and Adjusted Hazard Ratios of Contraceptive Discontinuation 100 Days Later (n=2,267)

Models of Method Information Indicators	HR (95% CI)	AHR^a^ (95% CI)
**Model I: MII**		
<3 questions	Reference	Reference
All 3 questions	0.59*** (0.42, 0.80)	0.65* (0.47, 0.91)
**Model II: MIIplus**		
<4 questions	Reference	Reference
All 4 questions	0.48*** (0.33, 0.68)	0.53*** (0.37, 0.76)
**Model III: MIIplus variation**		
<3 questions of MII	0.57 (0.31, 1.06)	0.56 (0.30, 1.04)
MII	Reference	Reference
MIIplus	0.28*** (0.14, 0.55)	0.31*** (0.17, 0.61)

Abbreviations: AHR, adjusted hazard ratio; CI, confidence interval; HR, hazard ratio; MII, Method Information Index; MIIplus, Method Information Index including the question, “Were you told about the possibility of switching to another method if the method you selected was not suitable?”

* *P*≤.05; **P≤.01; ***P ≤.001.

a Adjusted for age, education, religion, number of living children, previous modern method use, method selected at enrollment, and state.

Model III shows the adjusted HRs for discontinuation using the MIIplus variation variable. Women who received all information included in MIIplus were 69% less likely to discontinue using a modern method 100 days later compared with women who received the information in the MII (adjusted HR=0.31; 95% CI=0.17 to 0.61). No difference was observed in discontinuation among women who received information on less than 3 components of the MII compared to the complete MII.

### Modern Method Continuation by Enrollment Method and MIIplus

**FIGURE 4:**
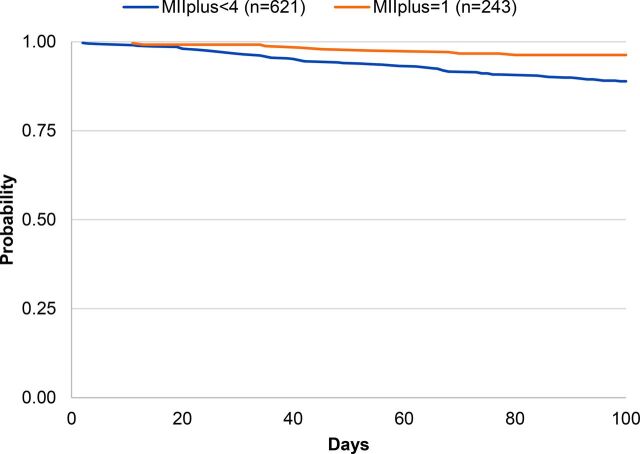
Kaplan-Meier Survival Curves of Contraceptive Continuation by MII With the Added Question About Method Switching (MIIplus) Among IUD Users at Enrollment Abbreviations: IUD, intrauterine device; MII, Method Information Index.

**FIGURE 5:**
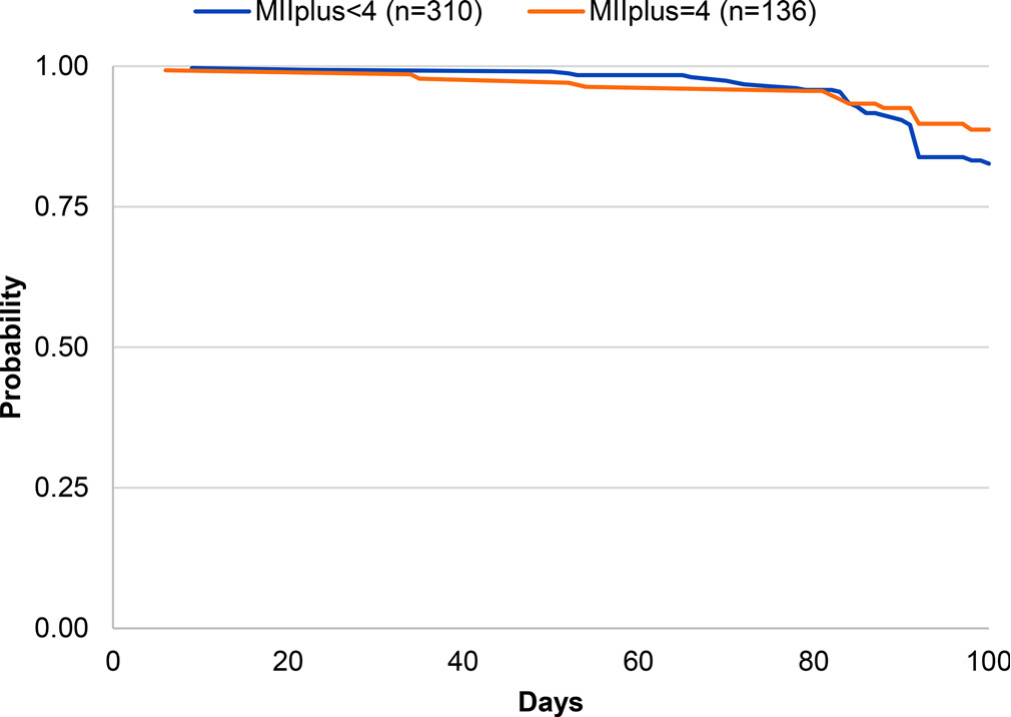
Kaplan-Meier Survival Curves of Contraceptive Continuation by MII With the Added Question About Method Switching (MIIplus) Among Injectable Users at Enrollment Abbreviation: MII, Method Information Index.

**FIGURE 6:**
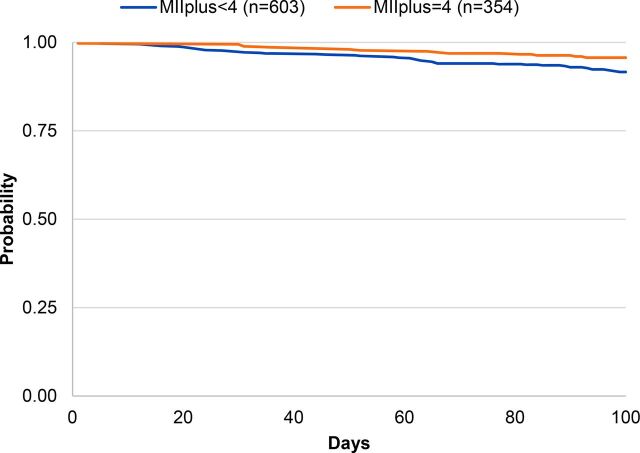
Kaplan-Meier Survival Curves of Contraceptive Continuation by MII With the Added Question About Method Switching (MIIplus) Among Pill Users at Enrollment Abbreviation: MII, Method Information Index.

Figure 4, Figure 5, and Figure 6 present the Kaplan-Meier survival curves for modern method continuation by MIIplus among IUD, injectable, and pill users at enrollment. The probability of continuing a modern contraceptive method at 100 days for IUD (both PPIUD and interval IUD), injectable, and pill users who received complete information in MIIplus was greater than among women who did not receive complete information in MIIplus. Continuation among IUD users at enrollment who received all information in MIIplus was 96% compared with 86% among IUD users who received information covered by MII but not about method switching (Figure 4). Similar relationships were seen for the injectable (Figure 5) and the pill (Figure 6), with injectable and pill users who received information in the MII and about the possibility of method switching being more likely to continue using 100 days later (89% for the injectable and 96% for the pill). In addition, for injectable users, most discontinuation occurred around the 100-day mark for women who received full information in the MIIplus and women who received information on less than all components included in MIIplus. Since the injectable is a 3-month method, active discontinuation would most likely occur around the time of reinjection.

## DISCUSSION

This study showed that women who report receiving full information included in MIIplus had a significantly lower risk of modern contraceptive discontinuation 3 months later compared with those who received full information included in the MII (Model III). The risk of discontinuation was not significantly different between those who received information on all aspects included in the MII compared with those who received information on less than those 3 items (Model III). In addition, the AIC was lower with MIIplus (Model II), suggesting the MIIplus model better fits the data compared with the MII (Model I).

The Kaplan-Meier survival estimates of MIIplus by method adopted at enrollment suggest that the MIIplus variable behaves similarly irrespective of method. The probability of modern contraceptive continuation among women who adopted the IUD, injectable, or pill and reported receiving complete information in MIIplus at method adoption was greater compared with those who had received information on less than 4 components included in MIIplus.

The MIIplus variable behaves similarly irrespective of method.

The possibility of switching methods may have been captured in the question, “Were you told what to do if you experience side effects?” In this study, a follow-up question (“What were you told?”) was asked of respondents who reported being counseled on what to do if they experienced side effects. No respondent reported that they were told about the possibility of switching. Furthermore, typically the question about what to do about side effects was only asked of respondents who reported that they were told about side effects. Information about the possibility of switching should be offered to all women and not necessarily linked to the experience of side effects because women discontinue contraceptive methods for numerous reasons, including cost and access. Also, the impact of physical side effects on women's lives and relationships could also affect decision making around method continuation,^7^ which may not necessarily be discussed in family planning counseling.

The MII has been explored in 2 other recent studies. A longitudinal study among family planning clients attending private franchise clinics in Pakistan and Uganda showed that women who did not receive full information according to the MII had a significantly increased hazard of contraceptive discontinuation 6 months after method adoption in Pakistan.^8^ The association was also observed among family planning clients in Uganda, but it was not significant. While the results presented here confirm these findings in public health facilities and through community-based distribution, the current study suggests that the observed relationship between the MII and discontinuation 3 months later may have been due to the fact that a large proportion of women also reported receiving information about method switching.

Based on the same datasets from Pakistan and Uganda, follow-up questions were asked of family planning clients after the MII questions to assess consistency in reporting.^9^ Follow-up questions included asking respondents to name another family planning method, specific side effects associated with the method received, and specific actions to take if a side effect was experienced. While the authors concluded that they observed significant decreases in the MII scores when they adjusted for consistency of the follow-up questions, consistency of 2 individual MII questions seemed high: (1) informed about other methods and naming another method, and (2) informed what to do if side effects are experienced and knowing the specific actions to take. Consistency was less likely when women were asked to name a specific side effect associated with the method they received. Additional work in this area is needed to validate a measure that accurately captures side effect counseling that women receive and the information that they take from it.

Another recent analysis compared measures of counseling on side effects captured in 3 data sources: (1) family planning clients' report of information received reported in the household survey of the DHS; (2) direct observations of client-provider interactions captured in the Service Provision Assessment (SPA); and (3) client exit interviews collected in the SPA.^10^ When comparing the SPA data sources, the author found that clients tended to overestimate information received in comparison to direct observations. While biases like courtesy bias and acquiescence bias may contribute to this overestimate, information that clients take away from their family planning consultations should be the focus of quality of care measurement because this information will likely influence their subsequent behaviors.

The results of the current study suggest that discontinuation of modern methods 100 days after initiation is lower for MIIplus compared with the MII alone in this sample population. MIIplus is a marker of good contraceptive counseling and includes information on an additional element of quality of care—continuity of use and follow-up. This information may help women continue to use modern contraception even if they discontinue their initially adopted method. For example, if a woman is told what to do if she experiences side effects but the recommended course of action is ineffective, then she may discontinue using contraception altogether because she did not know that she had the option to switch to a different method.

MIIplus is a marker of good contraceptive counseling and includes information on continuity of use.

While MIIplus is a better measure of quality of care compared with the MII (with the addition of another domain of quality of care) for voluntary family planning choice, it still falls short of reflecting all 4 domains of quality of care. MIIplus, however, could be used in cross-sectional and population-level surveys to obtain trends over time and across countries because it has been shown to predict lower levels of discontinuation compared with the MII. Additional measures that reflect all domains of quality of care have been proposed for routine monitoring of programs.^11^

As suggested elsewhere,^3^ adding the switching question for reversible contraceptive method users complements the question asked of sterilization users—whether they were told it was permanent—so that MIIplus could be based on 4 questions collected for all contraceptive users. The use of MIIplus as an indicator may encourage family planning programs and providers to move away from emphasizing method-specific continuation to continuation of any modern family planning (among women with a need for family planning). Furthermore, with the use of MIIplus in programs, women who find that their current method is no longer suitable at any time or for any reason may be more likely to switch to another contraceptive method.

### Limitations

The current study has several limitations. Most Indian women in this study who received full information according to their responses to questions in the MII also received information about the possibility of switching, suggesting that in India, providers have a tendency to routinely offer this information in family planning counseling. Future research in contexts in which this information may not be routinely given will help to further elucidate the relationship between being told about switching and modern contraceptive continuation.

A total of 432 respondents were excluded from these analyses because they were lost to follow-up, discontinued method use because they wanted to get pregnant, or had data inconsistencies. Logistic regression comparing these individuals with those who were included in these analyses (n=2,267) by respondent characteristics revealed no differences between these 2 groups for most characteristics (data not shown). However, 2 characteristics—religion and number of living children—were significantly different for respondents included and excluded from the analyses. Muslims compared with Hindus were significantly less likely to be excluded from these analyses, as were women with 2 or more children compared with those with 1 child. Because the final hazard models were adjusted for respondent characteristics, these differences were unlikely to have had a substantial effect on the conclusions.

Additional study limitations were that participants were not enrolled at the same time and the enrollment strategy varied by state and family planning method. IUD users were enrolled at public district hospitals, while eligible OCP users were identified through ASHAs at the community level. At the time of the study, the Indian government had committed to expanding method choice with the introduction of the injectable through public facilities, but rollout had not begun. Consequently, injectable users were recruited from NGO facilities. Thus, any differences observed in the quality of care received by clients for specific methods is likely due to the facility where the services were obtained rather than the method itself. Respondents in Haryana were enrolled in the study several months after enrollment began in Odisha. Future research should consider including the question about switching and replicating the analysis presented here to build the evidence base for the MIIplus.

Future research should consider including the question about switching to build the evidence base for the MIIplus.

## CONCLUSION

This study demonstrates that modern method discontinuation 3 months after method initiation is lower when women receive information in the MIIplus compared to the MII alone. By adding the question about method switching to the MII, MIIplus better reflects quality of care. Furthermore, shifting the outcome from method-specific discontinuation to modern method discontinuation allows the unit of analysis to reflect individual-level behaviors. Policymakers, programmers and donors who rely on the MII for national and sub-national estimates may be better served by using MIIplus to monitor quality of care in family planning services.

## Notes

**Peer Reviewed**

**Funding:** The Evidence Project is made possible by the generous support of the American people through the United States Agency for International Development (USAID) under the terms of cooperative agreement No. AIDOAA-A-13-00087.
